# Gravistimulation effects on *Oryza sativa* amino acid profile, growth pattern and expression of *OsPIN* genes

**DOI:** 10.1038/s41598-020-74531-w

**Published:** 2020-10-14

**Authors:** Muhammad Farooq, Rahmatullah Jan, Kyung-Min Kim

**Affiliations:** School of Applied Bioscience, Kyungpook National University, Daegu, 41566 Republic of Korea

**Keywords:** Molecular biology, Environmental sciences, Natural hazards

## Abstract

Gravity is an important ecological factor regulating plant growth and developmental processes. Here we used various molecular and biochemical approaches to investigate artificial and normal gravistimulation’s effect on the early growth stages of rice (*Oryza sativa* L.) by changing the orientations of Petri dishes. Rate of amino acid formation, root and shoot growth, and *OsPIN* expression was significantly higher under gravistimulation compared with the control. Clinostat rotation positively affected plant growth and amino acid profile. However, under normal gravity, vertical-oriented seedlings showed high amino acid levels compared with clinostat, 90°-rotated, and control seedlings. Similarly, seedling growth significantly increased with 90°-rotated and vertical orientations. Artificial gravity and exogenous indole-3-acetic acid induced *OsPIN1* expression in the roots, root shoot junction, and shoots of clinorotated seedlings. Phenyl acetic acid induced *OsPIN1* expression in the roots and root shoot junction of clinorotated seedlings but not in the shoot. The current study suggests that *OsPIN1* is differentially regulated and that it might be involved in the regulation of plant growth. Conversely, *OsPIN2* and *OsPIN3a* are gravity sensors and highly induced in the roots and root shoot junctions of vertical and 90°-rotated seedlings and play an important role in stress conditions. Thus, on exposure to gravity, hormones, and UV-C radiation, these genes are highly regulated by jasmonic acid, 6-benzylaminopurine and gibberellic acid.

## Introduction

Microgravity conditions impact biological processes such as graviperception and graviresponses. However, these processes are not yet fully understood. Although free-fall or parabolic flight can generate microgravity conditions, these methods do not provide sufficient time for researchers to study most morphogenetic and growth phenomena of plants. In this context, ground-based clinostats are instrumental. Continuous 360° rotation of plants on a clinostat eliminates a set direction of gravity and simulates plant growth under microgravity environments, which is useful to predict potential effects of spaceflight on biological specimens^1^. Continuous microgravity influences cellular components and cellular functions^2^. Gravistimulation applied through clinostat significantly increase the amino acid profile in plant tissue of pea, corn and rice^3^. Auxin is a phytohormone that plays a crucial role in the spatio-temporal aspects of plant growth and development. In addition to being essential in cell division, elongation, and differentiation, auxins also act as signals between cells, tissues, and organs. Clinostats may induce unusual growth in seedlings by preventing the accumulation of auxins in the roots or stem. Stems may then grow horizontally, instead of upward, and roots may grow toward the stem, rather than downward under the gravity effect^4^. Several reports have elucidated the effect of clinorotation on aspects of plant physiology, such as cytoskeletal function^5^, calcium distribution^6^, the cell cycle^7^, carbohydrate metabolism^8^ and protein expression^9^. The growth rates of many plant organs, such as lettuce hypocotyl, garden cress hypocotyl, and wheat coleoptile were increased under microgravity conditions^10,11^. Although the root growth rate of some species increased in the clinostat, the rate of root growth decreased when the level of plant hormones such as indole-3-acetic acid, abscisic acid, and zeatin reached supraoptimal levels^12,13^.

Indole-3-acetic acid (IAA), the main auxin in higher plants, maintains various developmental processes of plants, including growth direction, root and shoot branching, and vascular differentiation^14,15^. Auxins are considered to be synthesized in juvenile apical tissues, IAA is transported from these tissues in a unidirectional downward motion toward basally located plant tissues and organs, and by polar transport to the maturing stem and the leaves^16^. Auxin transportation is performed by auxin efflux and influx carrier proteins^17^. PIN-FORMED (PIN) efflux carrier proteins are plant-specific auxin transporters that play an important role in polar auxin transport and in establishing IAA concentration gradients^18^. IAA controls cell elongation, differentiation, and plant cell division in a concentration-dependent manner^19^. Plants mainly produce IAA from tryptophan by a two-step reaction that is spatio-temporally regulated^20,21^. The cellular IAA concentration is also regulated by various auxin-inactivated enzymes that catalyze methylation, oxidation, and conjugation with sugar or amino acids^22^. IAA is an important regulator of many plant developmental processes, including differentiation of vascular tissues, embryogenesis, apical dominance, root patterning, gravitropism, phototropism, and other physiological processes^23,24^. Phenyl acetic acid (PAA), previously found in seaweeds and vascular plants, is an auxinomimetic^22^ that has the same effects on plant growth as IAA. For example, PAA enhances the elongation of coleoptile segments of oats (*Avena sativa*) and the internodal segments of beans (*Phaseolus vulgaris*)^25,26^.

Plant growth and development is also affected by ultraviolet (UV)-A (320–400 nm), UV-B (280–320 nm) and UV-C (≤ 280 nm) radiation^27^. The level of UV-A is independent of ozone concentration because it is not attenuated by ozone, and it causes negligible damage to biological systems^28,29^. In contrast, UV-C is highly energetic and severely damaging to biological systems^30^. However, because the ozone layer and oxygen in the stratosphere actively absorb UV-C, it does not penetrate to the earth in appreciable amounts^31^. Exposure to UV radiation causes various morphological and physiological changes (deleterious and beneficial) in higher plants, with significant inter- and intra-differences observed among species^32^. UV-C radiation causes well-known deleterious effects, such as damage to DNA, proteins, lipids, cell membranes, and prevention of photosynthesis in plants^33^. However, UV-C can also have positive effects that enhance the quality of fruits and vegetables by increasing the concentration of health-promoting phytochemicals that extend shelf-life and, additionally, elicit stress-adaptive mechanisms in plants^27^. Both UV radiation and microgravity are hazardous to the biological system, the interaction between them are classified as an additive (neither sensation nor protection), synergistic (increase radiation effect under microgravity) or antagonistic (reduce radiation effect)^34^. UV-C-induced interplant communication likely plays an important role in ecological stability. Researchers have used co-culture experimental systems in a well-established model plant (*Arabidopsis thaliana*) to study UV-C-induced airborne interplant communication by alleviating transcriptional gene silencing in bystander plants under microgravity conditions in a two-dimensional rotating clinostat^35^.

In the current study, we performed experiments with rice (*Oryza sativa* L. cv. Nagdong) to test the hypothesis that microgravity and normal gravity differentially affect gene expression, amino acid profile, morphogenesis, and early stage plant growth. Amino acids are one of the key cellular components of plants that regulate different metabolic pathways by activating some enzymes, gene expression, and redox homeostasis. Therefore, the amino acid profile, growth pattern, and expression of *OsPIN* genes related to auxin efflux facilitation were measured in rice cv. Nagdong in supplemented agar medium under artificial (generated by clinostat) and normal gravity in (i) normal conditions, (ii) treatment with phytohormones, and (iii) UV-C radiation. The principal objective was to gain a better understanding of plant growth under altered gravity simulation on the ground and guide future space experiments (i.e., under real microgravity conditions).

## Materials and methods

Rice (*O. sativa* L. cv. Nagdong) plants were grown from seeds provided by the Plant Molecular Breeding Laboratory of Kyungpook National University, Republic of South Korea. There are 12 putative genes encoding auxin efflux transporters in the rice (*O. sativa* L.) genome. In this study, we measured the expression of three *PIN* genes (*OsPIN1, OsPIN2* and *OsPIN3a)* with accession numbers AF056027, AK101191 and AK063976, respectively. We designed primer sequences for each gene were using NCBI software (https://www.ncbi.nlm.nih.gov/): *OsPIN1* forward primer (FW) 5′GCGTCCGCACACCCAA3′, reverse primer (RV) 5′GCCAGTATCATCGCCACGTA3′; *OsPIN2* FW 5′AAGACCGTTGCGACATTTGC3′, RV 5′AGTACTCCCCTGAGCCCAAT3′; *OsPIN3a* FW 5′CCATGTACGGGCCATACTCC3′, RV 5′CAGGCCGCTGACTTCTGA3′. Additionally, *OsActin,* with accession number AB047313, was used as a control: (FW) 5′GGAACTGGTATGGTCAAGGC3′, (RV) 5′AGTCTCATGGATAACCGCAG3'.

### Plant material and growth conditions

Rice (*O. sativa* subsp. *japonica*) seeds, large-sized Petri dishes (150 × 20 mm), and plant agar (Duchefa Biochemie, Haarlem, Netherlands) were used for the experiments described herein. Rice seeds were germinated by soaking inside an incubator at 34 °C for 3 to 4 days. Before the soaking process, fungicides (500 µl) were added to 1 L of water. The rice seeds were kept separate in plastic bags previously punched with screws to provide holes allowing the aqueous fungicide solution to flow in and out to avoid fungal infection inside the incubator. The water was changed every 24-h. After 3 to 4 days, the germinated rice seeds were surface-sterilized with 5% (w/v) sodium hypochlorite for 5 min and then rinsed thoroughly with distilled water 3–4 times. The sterilized seeds were placed within a 1300 series A2 biological safety cabinet (Thermo Scientific). All steps of the experiment were performed on a clean shelf within the safety cabinet to avoid bacterial and fungal contamination.

For growing conditions under normal gravity, we used three Petri dish orientations: horizontal, vertical, and 90°-rotated. To stimulate artificial gravity and generate the microgravity-like effect, we used one-axis clinostat. The clinostat rotated continuously in an anti-clockwise direction at a speed of 10 rpm, and the distance from *O. sativa* seedlings to the rotating center was maintained at approximately 5 cm. Thus, the seedlings were subjected to a modeled microgravity of approximately 5.59 × 10^–3^ g. Horizontal (control), vertical, and 90°-rotated Petri dishes were kept under 1 g (Table 1). Depending on experimental requirements, rice seedlings were grown under different orientations along with clinostat for 7 days in a growth chamber at 28/25 °C (day/night) with a 16/8 h light/dark under 50% relative humidity^36^.

**Table 1:** Different orientations used for gravity stimulation and clinostat.

Type of plant	Time of gravity	Rotation speed (rpm)	Rotational axis angle	Rotation direction	Orientation
Rice cv. Nagdong	7 days	×	×	×	Horizontal (control)
	7 days	×	×	×	Vertical
	7 days	×	×	×	90° Rotated
	7 days	10	90	Anti-clockwise	Clinostat

### Plant agar media

Agar medium (Duchefa Biochemie) was prepared by dissolving agar (15 g/L) in double-distilled water. Then, the solution was mixed on a magnetic stirrer and autoclaved at 121 °C for 40 min. When the mixture had cooled to 60 °C, replicates were dispensed into Petri dishes for solidification at 37 °C. We placed the germinated rice seeds of cv. Nagdong onto the surface of solidified agar in each Petri dish and subjected them to gravistimulation, as shown in Table 1. After 7 days, we analyzed the amino acid contents, root and shoot length, and the relative expression of selected *OsPIN* genes.

### Amino acid analysis

**Figure 1:**
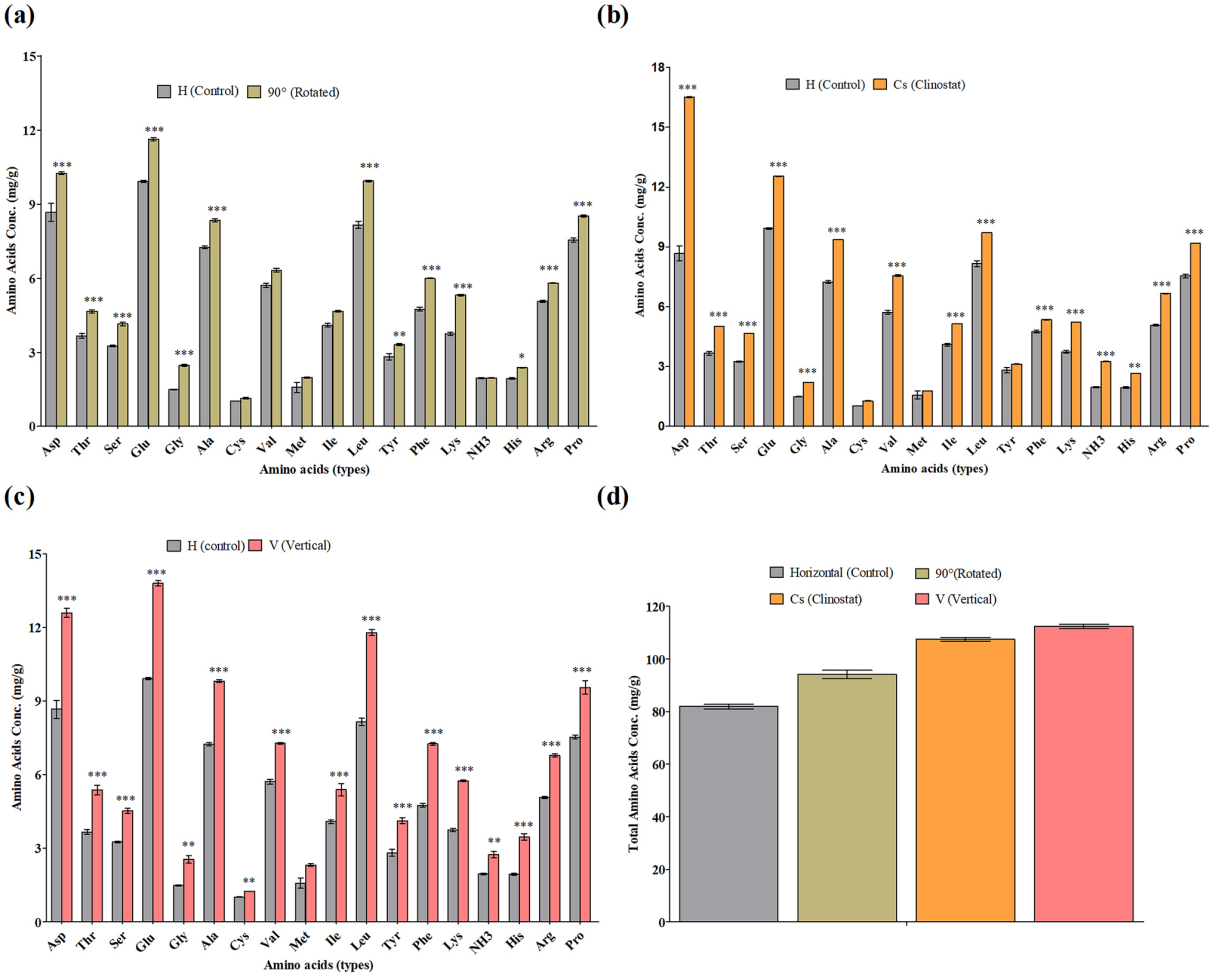
Amino acid concentrations (mg/g) in rice seedlings (*Oryza sativa* L. cv. Nagdong) grown in supplemented agar medium under normal and artificial gravistimulation. Total amount of amino acids (mg/g) in rice seedlings grown under (**a**) horizontal (control) 90°-rotated orientation, (**b**) clinostat rotation, (**c**) vertical orientation and (**d**) the various orientations as compared to horizontal (control). The vertical bars indicate mean ± standard error of the mean. Statistical analysis was calculated by the Bonferroni post-test two-way RM ANOVA method. Asterisks in the vertical bar indicate a significant difference at ^*^p < 0.05, ^**^p < 0.01, ^***^p < 0.001 compared to controls.

We performed amino acids analysis of rice seedlings grown in the differently oriented Petri dishes, along with the samples grown on the clinostat, using the method previously described^37^. Gently-ground whole plant samples (100 mg) were hydrolyzed under vacuum in 6 N HCl at 110 °C, followed by 80 °C for 24 h, respectively. The dried residues were homogenized in 0.02 N HCl and passed through a 0.45-µm filter prior to injection into the Ezchrom Elite for Hitachi L-8900 amino acid analyzer (Hitachi). Ammonia was excluded because it may contain ammonia gas, which is generated during the analysis. The total amino acid content of the composition, minus the ammonia nitrogen, is listed (Fig. 1d).

### Phytohormone treatment

Phytohormones are signaling molecules that regulate many growth and developmental processes in plants, but they normally occur in extremely low concentrations. Therefore, we germinated rice seeds on agar media containing 10 µM each of the following phytohormones: indole-3-acetetic acid (IAA), phenylacetic acid (PAA), jasmonic acid (JA), 6-benzylaminopurine (6-BAP) and gibberellic acid (GA). The agar media was prepared as described above. Then, Petri dish replicates were placed under different orientations and on the clinostat. After 7 days, we measured the root shoot length and the expression of selected *OsPIN* genes through quantitative RT-PCR in the roots, root shoot junction, and shoots of rice seedlings under gravistimulation.

### Irradiation with UV-C under the treatment of various plant hormones

The germinated rice seeds were immediately irradiated with UV-C radiation for 30 min at a distance of 100 cm from the UV source in a 1300 Series A2 Type A2 biological safety cabinet (230 V, 50/60 Hz; 2 W). Afterwards, the rice seeds were placed in three replicates of Petri dishes containing 10 µM of the various phytohormones (IAA, PAA*,* JA*,* 6BAP, and GA) and were subjected to gravistimulation. After 7 days, we measured root growth, shoot growth, and relative expression of selected *OsPIN* genes in various parts of the rice seedlings.

### Quantitative RT-PCR analysis

After 7 days, we extracted total RNA from the various parts of the rice seedling grown under normal and artificial gravity using the RNeasy plant mini kit (Qiagen, Germany) according to the specified instructions. We used a NanoDrop 2000 spectrophotometer (Thermo Scientific, Wilmington, DE, USA) to measure RNA concentrations. For first-strand cDNA synthesis, the qPCRBIO cDNA synthesis kit and 400 ng of total RNA were used. For quantitative RT-PCR, we used the Eco Real-Time PCR system (Illumina, Inc., San Diego, CA, USA), 2X qPCRBIO SyGreen (www.pcrbio.com, London, UK), and primers specific for the selected genes. *OsActin* was used as an internal reference gene for normalization.

### Statistical analyses

The experiment comprised three treatments, each with three replicates and 27 seedlings. The experiment was repeated three times and the combined data is presented as the mean ± standard error of the mean. The data were divided into three groups: normal conditions, phytohormone treatment, and UV-C radiation with phytohormone treatment. Statistical comparisons were calculated by the Bonferroni post-test two-way RM ANOVA method. Asterisks in the vertical bar indicate a significant difference at ^*^p < 0.05, ^**^p < 0.01, ^***^p < 0.001 compared to controls. Figures were made using Graph Pad Prism, version 5.0 (Graph Pad software Inc., San Diego, California, USA).

## Results

### Amino acids profile under gravistimulation

Examination of the effect of artificial and normal gravity stimulation revealed that different amino acid levels were higher in seedlings in Petri dishes placed under vertical orientation, clinostat, and 90°-rotated orientations (Fig. 1a–c) as compared to controls (plants grown under horizontal orientation). There was a higher proportion of Asp, Glu, Ala, Leu, and Pro amino acids under gravistimulation relative to other amino acids. Other amino acids, such as Thr, Ser, Gly, Cys, Val, Met, Iso, Tyr, Phe, Lys, His, and Arg, were detected in lower proportions under gravity stimulation, but their concentrations were still higher than those in the control horizontal orientation. In this study, we examined 18 out of 20 amino acids. Among the various orientations, aspartic acid was detected at higher concentrations in clinostat seedlings, whereas the glutamic acid concentration, which was the amino acid found at the second-highest concentrations in all orientations, was higher in vertical-oriented seedlings.

We also determined the total amount of amino acids by summing all 17 amino acids (in mg/g), without ammonia, from each orientation. The total amino acid concentration was higher in vertical-oriented seedlings as compared to clinostat, 90°-rotated, and horizontal (control) orientation (Fig. 1d). These results show that the amino acid concentrations under microgravity-like condition, and as well as in different orientations under normal gravity, are significantly enhanced in plants grown in agar media. Under normal gravity conditions, the vertical-oriented rice seedlings had higher proportions of Thr, Glu, Gly, Ala, Met, Iso, Leu, Tyr, Phe, Lys, His, Arg, and Pro compared to control, 90°-rotated, and clinostat rotation (Fig. 1c).

### Direction and length of roots and shoots

**Figure 2:**
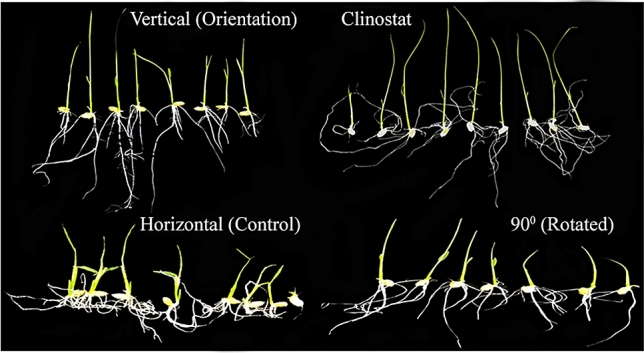
Phenotypic characteristics of 7-day-old rice seedlings (*Oryza sativa* L. cv. Nagdong) grown in supplemented agar medium under gravistimulation.

**Figure 3:**
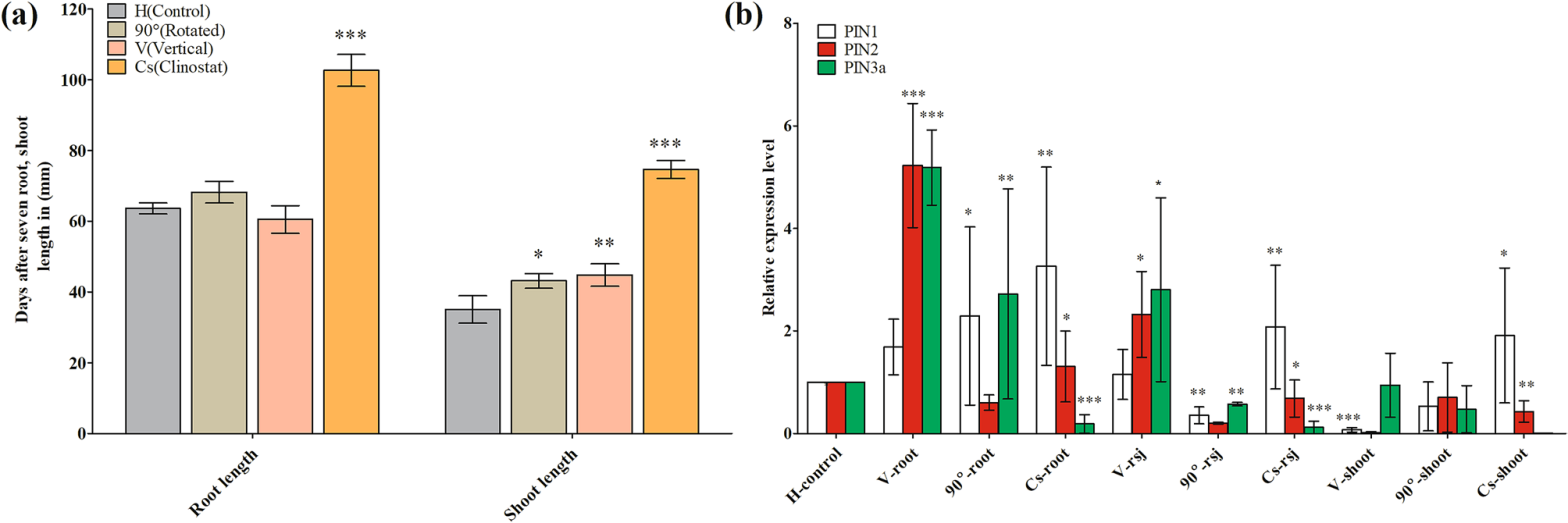
Root length, shoot length, and relative expression of selected *OsPIN* genes in rice seedlings (*Oryza sativa* L. cv. Nagdong) grown in supplemented agar medium under gravistimulation. (**a**) Roots and shoots length (**b**). Relative expression of selected *OsPIN* genes in the root, root shoot junction (rsj), and shoot. The experiment was repeatedly conducted under controlled greenhouse conditions without any treatment. Vertical bars indicate mean ± standard error of the mean (*n* = 3). Asterisks in the vertical bar indicate significant difference at ^*^p < 0.05, ^**^p < 0.01, ^***^p < 0.001 compared to controls.

In this study, we found different orientation- and microgravity-like condition dependent growth patterns in rice seedlings. In clinorotated rice seedlings (10 rpm anti-clockwise rotation), the stems grew horizontally downward toward the direction of the earth while the roots grew upwards. In 90°-rotated, vertical and horizontal control orientations, the roots and shoots grew normally: root growth downward and shoot growth upward toward the direction of light. In terms of phenotypic appearance, clinostat, vertical, and 90°-rotated rice seedlings showed a higher growth rate as compared to horizontal controls (Fig. 2). We also investigated the root length and shoot length of rice seedlings. It was noted that under normal gravity and artificial gravity, there was no significant difference in root length, except in the clinostat group. Conversely, the shoot length was significantly different in 90°-rotated and vertical orientations as compared to the horizontal control. Similarly, shoots growth in the clinostat group was significantly longer as compared to the horizontal control orientation (Fig. 3a). We also used RT-PCR to determine the expression of selected *OsPIN* genes in roots, root shoot junction, and shoots of rice seedlings. Under normal and artificial gravity, we found that *OsPIN3a* and *OsPIN2* genes were highly induced in the roots, root shoot junction, and shoots of vertical and 90°-rotated seedlings. Conversely, the *OsPIN1* gene was highly induced in the roots, root shoot junction, and shoots of clinorotated seedlings which suggests a prevention of hormone (auxin) accumulation on one side of the roots or stem as compared to horizontal controls (Fig. 3b).

### Effect of phytohormones on root and shoot growth under gravistimulation

**Figure 4:**
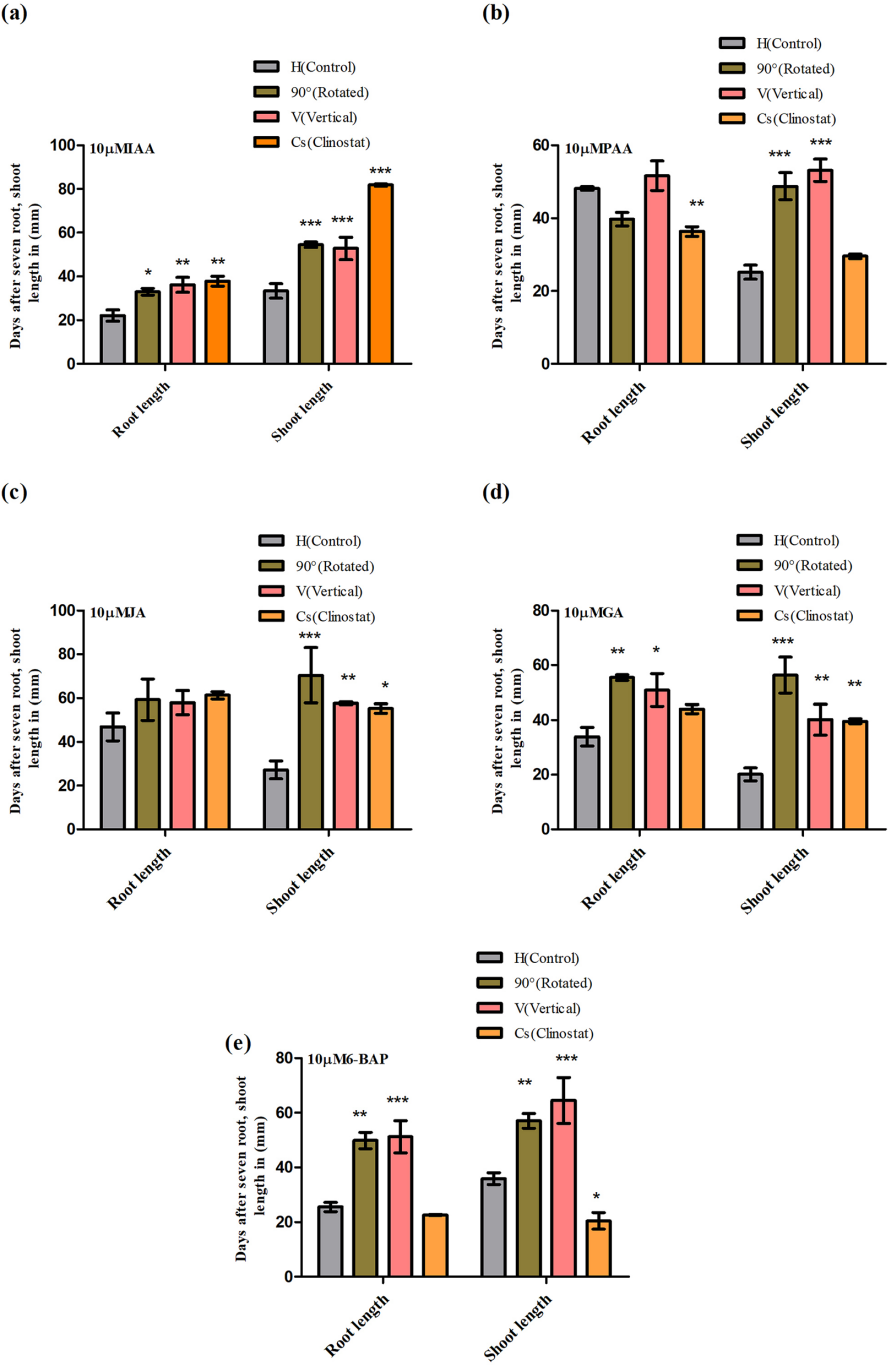
Rice (*Oryza sativa* L. cv. Nagdong) root length and shoot length after gravistimulation under exogenous phytohormone treatment. Indole-3-acetic acid (IAA) (**a**), phenyl acetic acid (PAA) (**b**) jasmonic acid (JA) (**c**), gibberellic acid (GA) (**d**), and 6-benzylaminopurine (6-BAP) (**e**) were used at 10 µM in agar medium. The vertical bars indicate mean ± standard error of the mean. Statistical analysis was calculated by the Bonferroni post-test two-way RM ANOVA method. Asterisks in the vertical bar indicate significant difference at ^*^p < 0.05, ^**^p < 0.01, ^***^p < 0.001 compared to controls.

We found that each hormone differentially regulated root and shoot growth under gravistimulation (Fig. 4). Under normal and artificial gravity conditions, both IAA and PAA hormones highly accelerated shoot growth while root growth was significantly increased by IAA instead of PAA. However, under clinostat rotation, PAA had no significant impact on shoot growth when compared to controls (Fig. 4a,b). Similarly, we observed no difference in root growth with exogenous JA and GA, except in 90°-rotated and vertical orientation, wherein the root growth was significantly increased by GA. Conversely, JA and GA enhanced shoot growth in 90°-rotated, vertical orientation, and clinorotated seedlings (Fig. 4c,d). In addition, 6-BAP caused a substantial decrease in both root and shoot growth in clinostat conditions, but enhanced root and shoot growth in 90°-rotated and vertical orientation compared to controls (Fig. 4e).

### Effect of UV-C radiation on root and shoot growth under phytohormone treatment and gravistimulation

**Figure 5:**
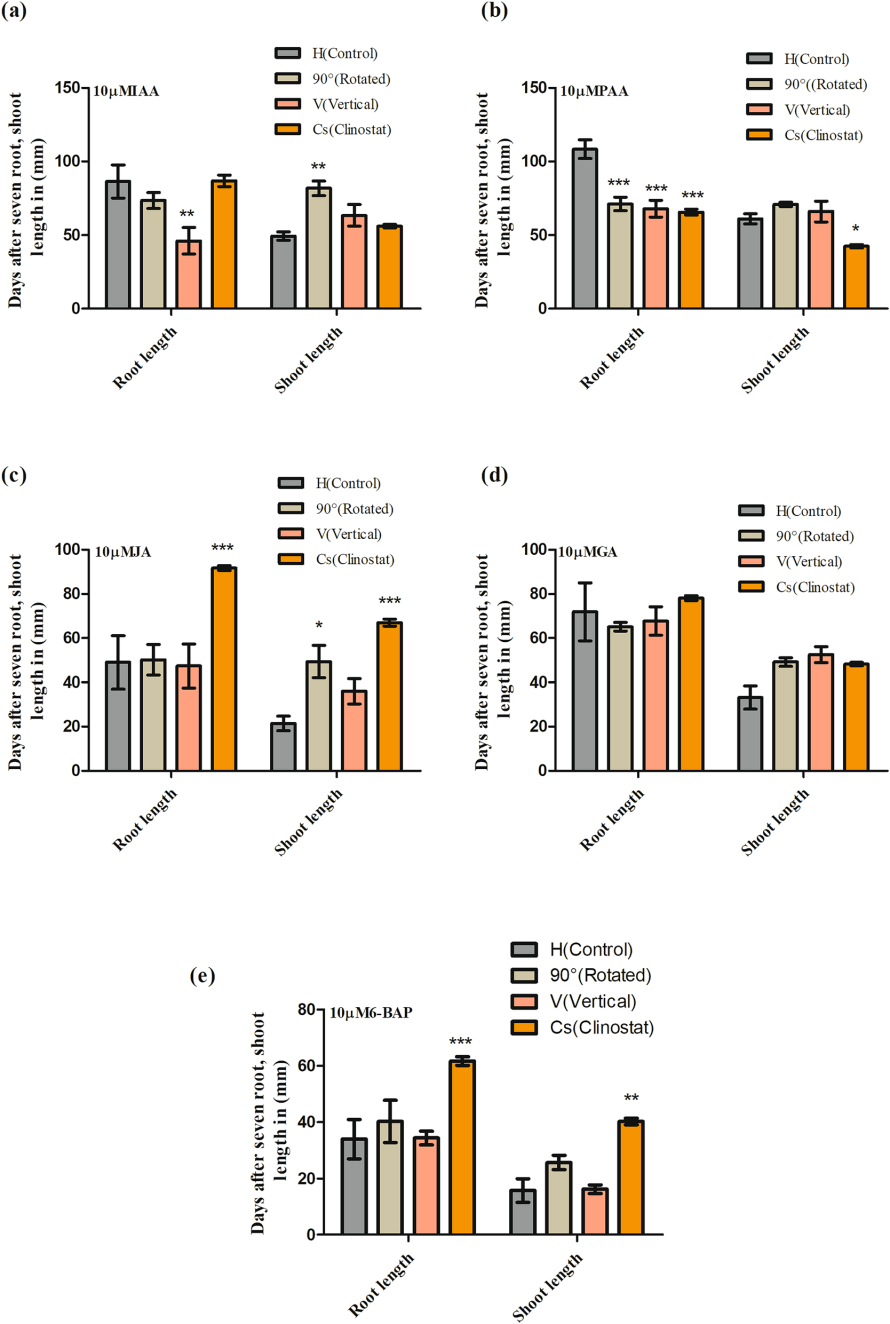
Rice (*Oryza sativa* L. cv. Nagdong) root and shoot length following exposure to UV-C radiation, under exogenous phytohormone treatment and gravistimulation. Phytohormones indole-3-acetic acid (IAA) (**a**), phenyl acetic acid (PAA) (**b**), jasmonic acid (JA) (**c**), gibberellic acid (GA) (**d**), and 6-benzylaminopurine (6-BAP) (**e**) were used at 10 µM in agar medium. Vertical bars indicate mean ± standard error of the mean. Statistical analysis was calculated by the Bonferroni post-test two-way RM ANOVA method. Asterisks in the vertical bar indicate significant difference at ^*^p < 0.05, ^**^p < 0.01, ^***^p < 0.001 compared to controls.

Next, we studied the effect of UV-C radiation combined with plant hormone treatment on rice seeds under different gravity conditions. We found that UV-C radiation differentially stimulated root and shoot growth of rice (Fig. 5). The use of IAA led to similar root lengths among the 90°-rotated, clinostat, and controls but not in the vertical orientation group. On the contrary, the shoot length in 90°-rotated orientation was significantly higher than that of controls (Fig. 5a). Similarly, PAA significantly decreased root length growth compared to controls, and decelerated shoot growth in clinostat seedlings (Fig. 5b). Under UV-C radiation, JA proved to be an effective enhancer of root and shoot growth in clinostat conditions (Fig. 5c). Similarly, it also enhanced shoot growth in 90°-rotated orientation relative to controls (Fig. 5c). Regarding JA, the root growth of 90°-rotated and vertical orientations were similar to controls (Fig. 5c). Conversely, after UV-C absorption, GA had no effect on root and shoot growth in 90°-rotated, vertical orientation, and clinostat conditions compared to controls (Fig. 5d). After UV-C absorption, 6-BAP treatment highly increased root and shoot growth in the clinostat group, but the 90°-rotated and vertical orientation groups did not differ from controls (Fig. 5e).

### Effect of gravistimulation and phytohormones on OsPIN gene expression

**Figure 6:**
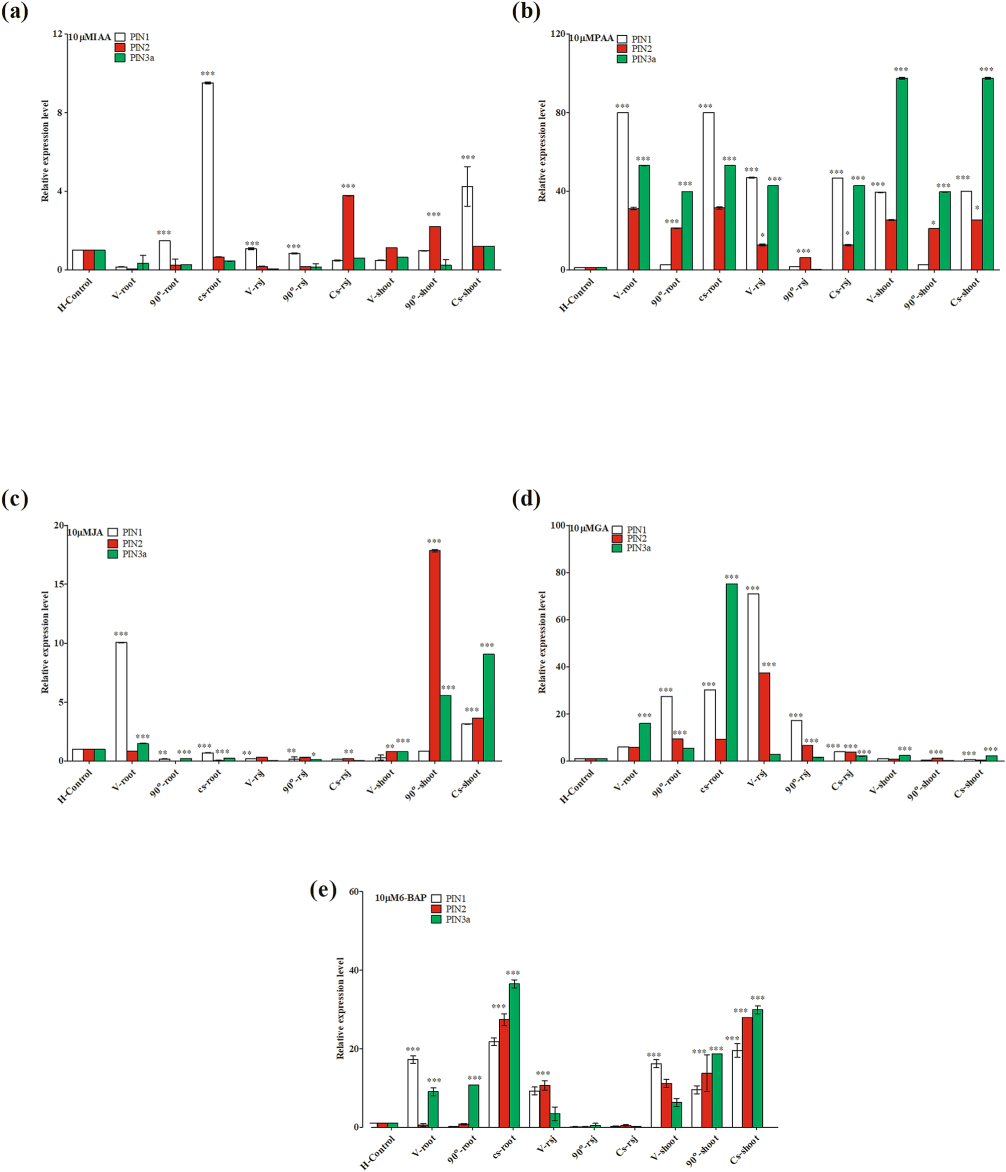
Quantitative RT-PCR analysis of selected *OsPIN* genes in rice (*Oryza sativa* L. cv. Nagdong) under exogenous phytohormones treatment and gravistimulation. Phytohormones indole-3-acetic acid (IAA) (**a**), phenyl acetic acid (PAA) (**b**), jasmonic acid (JA) (**c**), gibberellic acid (GA) (**d**), and 6-benzylaminopurine (6-BAP) (**e**) were used at 10 µM in agar medium. Vertical bars indicate mean ± standard error of the mean (*n* = 3). Statistical analysis was calculated by the Bonferroni post-test two-way RM ANOVA method. Asterisks in the vertical bar indicate significant difference at ^*^p < 0.05, ^**^p < 0.01, ^***^p < 0.001 compared to controls.

Self- and mutual-regulation of plant hormones plays an essential role in developmental and environmental processes. In this study, we investigated the effects of both gravity and plant hormones on the expression of three selected *OsPIN* genes in different parts of rice seedlings (Fig. 6). We observed that under gravistimulation, IAA strongly induced *OsPIN1* expression in the roots of 90°-rotated and clinorotated seedlings and in the shoots of clinorotated seedlings. The *OsPIN2* gene was induced in the root shoot junction of clinorotated seedlings (Fig. 6a). Meanwhile, PAA highly induced *OsPIN1* in the root and root shoot junction of clinostat and vertical-oriented seedlings, and induced *OsPIN3a* in the shoot of vertical, 90°-rotated, and clinorotated seedlings (Fig. 6b). JA treatment induced *OsPIN1* in the root of vertical-oriented seedling, while a high expression of *OsPIN2* and *OsPIN3a* genes were observed in the shoots of 90°-rotated, and clinorotated seedlings (Fig. 6c). Similarly, *OsPIN1*, *OsPIN2,* and *OsPIN3a* were highly induced by GA in the root of vertical, 90°-rotated, and clinorotated seedlings, while it induced high levels of *OsPIN1* and *OsPIN2* genes in the root shoot junction (Fig. 6d). 6-BAP treatment mainly induced *OsPIN1* genes in the roots and shoots of vertical orientations, while in the roots and shoots of 90°-rotated and clinorotated seedlings, it increased expression of *OsPIN3a* and *OsPIN2* genes (Fig. 6e).

### Effect of UV-C radiation and phytohormones on OsPIN gene expression in rice under gravistimulation

**Figure 7:**
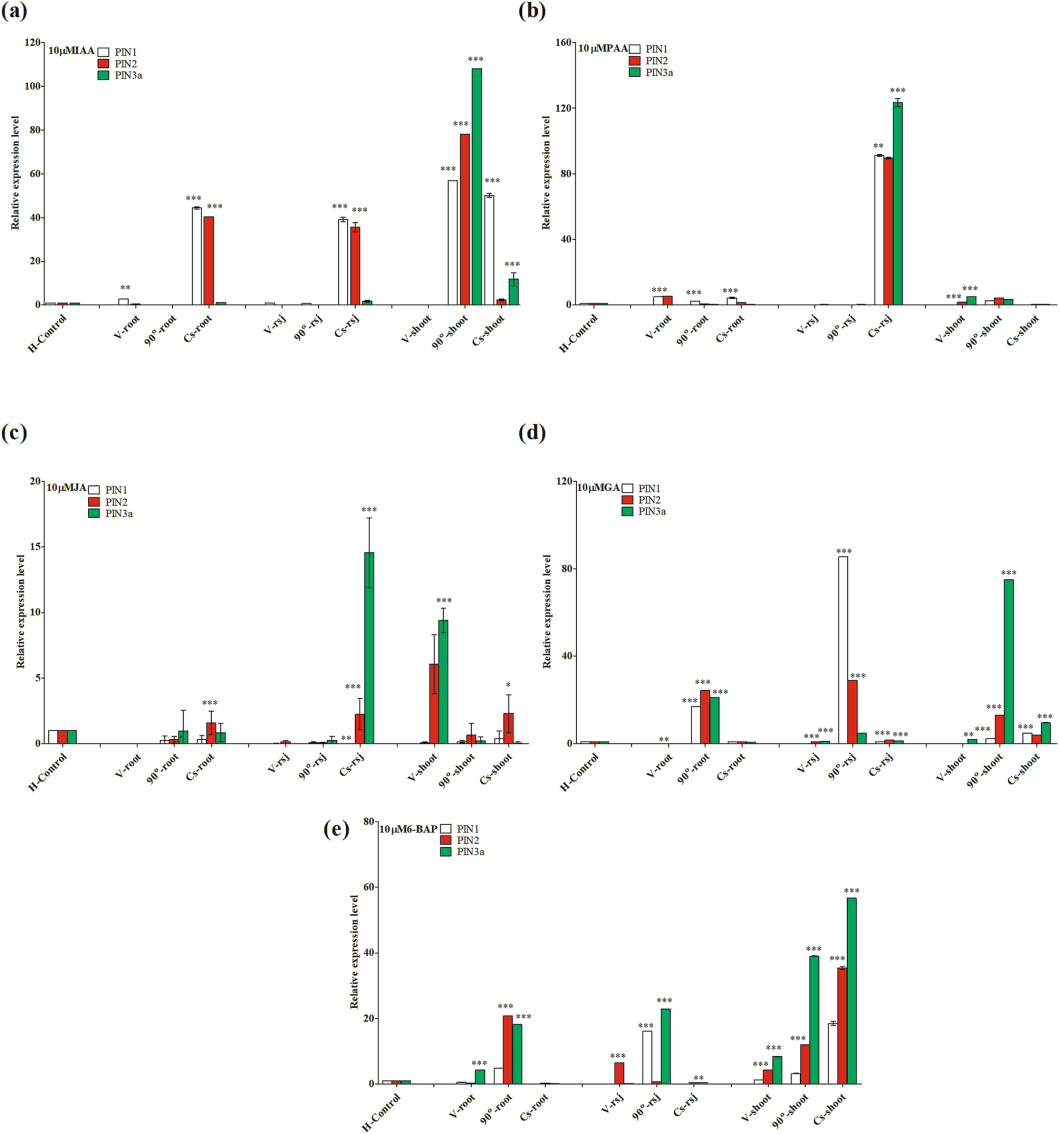
Expression of selected *OsPIN* genes in rice (*Oryza sativa* L. cv. Nagdong) seedlings subjected to UV-C radiation, phytohormones, and gravistimulation. Phytohormones indole-3-acetic acid (IAA) (**a**), phenyl acetic acid (PAA) (**b**), jasmonic acid (JA) (**c**), gibberellic acid (GA) (**d**), and 6-benzylaminopurine (6-BAP) (**e**) were used at 10 µM in agar medium after UV-C radiations. Vertical bars indicate mean ± standard error of the mean (*n* = 3). Statistical analysis was calculated by the Bonferroni post-test two-way RM ANOVA method. Asterisks in the vertical bar indicate significant difference at ^*^p < 0.05, ^**^p < 0.01, ^***^p < 0.001 compared to controls.

UV irradiation, along with phytohormone treatment, differentially regulated *OsPIN* gene expression under gravistimulation*.* After UV-C absorption, IAA highly induced *OsPIN1* in the roots, root shoot junction, and shoots of clinorotated seedlings, while in 90°-rotated seedlings, we observed a high expression of *OsPIN2* and *OsPIN3a* in the shoots (Fig. 7a). In contrast, PAA induced *OsPIN1* and *OsPIN2* expression in the roots of vertical, 90°-rotated, and clinorotated seedlings. However, in the root shoot junction of clinostat, and shoots of vertical and 90°-rotated seedlings, *OsPIN3a* was highly induced by PAA (Fig. 7b). In regards to JA treatment after UV-C absorption, this phytohormone highly induced *OsPIN3a* in the root shoot junction of clinorotated seedlings, as well as the shoots of vertical-oriented seedlings (Fig. 7c). GA treatment induced *OsPIN2* and *OsPIN3a* in the roots and shoots of 90°-rotated, clinorotated, and vertical-oriented seedlings. However, GA induced *OPIN1* expression in the root shoot junction of 90°-rotated seedlings (Fig. 7d). Similarly, after UV-C absorption, exogenous 6-BAP mainly induced *OsPIN3a* and *OsPIN2* in the roots, root shoot junction, and shoots of clinorotated, 90°-rotated, and vertical-oriented seedlings. In the roots and the root shoot junction of clinorotated seedlings, *OsPIN1, OsPIN2* and *OsPIN3a* were downregulated in comparison to controls (horizontal orientation) (Fig. 7e).

## Discussion

In a recent study, clinostat rotation positively affected the concentration of amino acids in rice under Murashige–Skoog medium supplementation^3^. The results of the present study demonstrate that the essential amino acids of rice grown under artificial and normal gravity are also enhanced in agar medium. We found that vertical-oriented Petri plates, in which seeds were placed in vertical position under normal gravity in agar medium, highly enhanced amino acids contents in comparison to clinostat, 90°-rotated, and control seedlings (Fig. 1c).

Earlier studies reported the effect of gravity on cell wall properties in plant organs and demonstrated that the rigidity (mechanical strength) of cell walls increased under conditions of hyper-gravity (i.e., gravitational forces > 1 × *g*) produced by centrifugal acceleration^38^. It has been noted that under microgravity conditions in space, roots elongated either in the direction of the seed^39,40^ or in irregular directions^41^. It was also reported that under microgravity conditions, the growth of roots and shoots was stimulated whereas lateral expansion was suppressed. Changes in cell wall properties account for these growth pattern modifications under microgravity conditions^2^. Microgravity may influence the activity of mechanoreceptors, which could cause an enhancement in growth of rice roots in space^42^. In earlier work under microgravity condition, stem lengths were increased, but there was no effect on root lengths^43^. However, in the present experiment, we observed that both root and shoot growth was stimulated by clinostat rotation at 10 rpm for 7 days as compared to normal gravity. We also observed that with clinostat rotation, the roots of rice seedlings grew randomly upwards toward the direction of light and the shoot grew downwards, whereas in the other orientations, the direction of root and shoot growth was normal. We found that normal gravity did influence shoot growth, but not root growth in the 90°-rotated and vertical-oriented rice seedlings. (Figs. 2 and 3a).

Among the seven plant hormones, auxins and GA are the strongest accelerators of shoot growth and decelerators of root growth, whereas, abscisic acid, ethylene, and JAs are suppressive hormones for both shoot and root growth^44,45^. In addition to IAA, other endogenous auxins have been identified in plants^46^. PAA is an active auxin found in various plant species, as well as in bacteria, although PAA has a much weaker effect than IAA and its role during plant development is unclear. The chlorinated form of IAA (4-chloroindole-3-acetic acid [4-Cl-IAA]) is found in great abundance in pea seeds^47^ and plays a crucial role during fruit development. Indole-3-butyric acid (IBA) has also been suggested to function as an internal IAA precursor, forming IAA via peroxisomal β-oxidation and playing a role during seedling development^48^. Whether IBA also has an auxin-like effect is still not fully understood, although genetic evidence of its concentration suggests that this is not the case^49^. IAA is present in free (bioactive) and conjugated (stored) forms. Local free IAA contributes to the regulation of plant growth and development^50,51^. In the current study, IAA had a strong effect on root and shoot growth. IAA increased the root and shoot growth in both normal and artificial gravity. PAA also increased shoot growth in 90°-rotated and vertical orientations, and root growth of 90°-rotated and vertical orientation seedlings was found to be similar to controls except in the clinostat group, which was significantly decreased by PAA. Similarly, GA, JA and 6-BAP enhanced both root and shoot growth of 90°-rotated, vertical-orientation, and clinostat seedlings. However, 6-BAP strongly decelerated both root and shoot growth in clinostat (Fig. 4a–e).

Synthetic auxin (Dichlorophenoxyacetic acid, 2,4-D) is destroyed by radiation between 340 and 390 nm, and light converts 2,4-D to 2,4-dichlorophenol, which is poisonous to cells^52^. In UV-irradiated seedlings, growth can be triggered by gibberellins^53^. The plant hormone ethylene, which changes elongation to radial growth, is produced to a greater extent in UV-B irradiated than non-irradiated plants^54^. The current study showed that after UV-C irradiation, JA and 6-BAP both stimulated root and shoot growth under artificial gravity conditions, whereas PAA significantly reduced root growth under both normal and artificial gravity. In addition, IAA and GA accelerated root and shoot growth equally, except in vertical orientation roots that were significantly reduced by IAA, and in 90°-rotated orientation shoots that were significantly increased by IAA under normal gravity. However, clinorotated seedlings responded well to JA, and exhibited a significant increase in root and shoot growth (Fig. 5a–e). A previous study revealed that an exogenously spray of 6-BAP reduced the stem length of flowering plants^55^.

The rice genome contains 12 reported *PIN* genes^56^, including four *PIN1* genes (named *OsPIN1a–1d*), *OsPIN2*, three *PIN5* genes (*OsPIN5a–c*), *OsPIN8*, and three monocot-specific *PIN* genes (*OsPIN9, OsPIN10a* and *OsPIN10b*). It is suggested that root-ward *PIN2* and *PIN1* are particularly sensitive to shoot-ward relocation^57^. The *PIN3*, *PIN4* and *PIN7* genes appear to function in tropisms, root meristem patterning, and laying the foundation of embryonic polarity, respectively^58–60^. Changes in *PIN2* genes display agravitropic root growth phenotypes^61–63^.

In response to gravity, cucumber seedlings have the potential to develop a peg on each side of the transition zone whether the seeds are germinated in vertical position with the radicle pointing downward or under microgravity conditions in space. Additionally, peg formation on the upper side of the transition zone is suppressed when the seedlings are grown horizontally on the ground^64^. Auxin plays a crucial role in gravimorphogenesis by establishing lateral placement of peg formation in the transition zone^65,66^.

In rice, the *OsPIN1* gene actively promotes auxin transport^67^, and *OsLazy1* inhibits auxin transport^68^. Among the entire set of 12 putative *OsPIN* genes, we focused our study on *OsPIN1, OsPIN2,* and *OsPIN3a* and monitored their relative expression under the effect of gravity and different plant hormones. Our results revealed that exogenous IAA highly induced *OsPIN1* expression in the root and shoot of clinorotated seedlings. In addition, the *OsPIN1* gene is also induced by PAA in the root of vertical and clinorotated seedlings, while in the shoot, there was a high expression of *OsPIN3a*. Similarly, JA induced the *OsPIN1* gene only in the root of vertical-rotated seedlings, while *OsPIN2* and *OsPIN3a* genes were highly expressed in the shoot of 90°-rotated, and clinostat seedlings. GA induced the expression of *OsPIN1* in the root shoot junction of vertical, 90°-rotated, and clinostat seedlings. However, in the root region, *OsPIN1* and *OsPIN3a* were highly expressed. Similarly, exogenous 6-BAP induced the expression of *OsPIN2* and *OsPIN3a* in both roots and shoots of clinostat seedlings with markedly decreased shoot growth (Fig. 6a–e).

In *Arabidopsis* roots, gravistimulation induces *AtPIN3* and *AtPIN7* upregulation in the plasma membrane of the lower side of the columella cells^69^. This gravity-modulated upregulation of *AtPIN3* and *AtPIN7* may contribute to alterations due to gravity in the lateral auxin transport system in roots given that the endodermis is recognized to sense gravity in the shoot^70^. The endodermis in the transition zone of cucumber seedlings has also been identified as a gravity sensor^71^. *CsPIN1*, which encodes a *PIN* auxin efflux facilitator, was previously identified in the endodermis of the transition zone of cucumber^72^. In the current study, we also identified a high expression of *OsPIN3a* and *OsPIN2* in the roots, root shoot junction, and shoot of rice seedling under normal gravity stimulation. Conversely, we detected a high expression of *OsPIN1* in the roots, root shoot junction, and shoots of the clinorotated seedlings under artificial gravity stimulation (Fig. 3b).

UV irradiation can not only alter or destroy amino acid residues but can also lead to inactivation of entire proteins and enzymes^73^. *PyroA* and *Ubq3* genes are upregulated by UV-B irradiation^74^. UV-B also activates the production of reactive oxygen species^75,76^ and antioxidant defenses^77,78^. Among other effects, UV-B reduces stomatal density in rice cultivars^79^. Although UV-B alters plant morphology, in many instances, UV-B positively influences plant defense against biotic stress^80^. Gene expression is also altered when plants are exposed to UV-B irradiation^81^. In our study, we examined the effect of UV-C radiation, exogenously applied phytohormones, and gravistimulation on *OsPIN1*, *OsPIN2,* and *OsPIN3a* genes in rice. We found that *OsPIN3a* and *OsPIN2* were highly induced by IAA in the shoot of 90°-rotated seedlings. Similarly, *OsPIN3a* was induced by PAA in the root shoot junction of clinorotated seedlings. In the same manner, treatment with JA, GA and 6-BAP plant hormones after UV-C radiation induced the expression of *OsPIN3a* and *OsPIN2* genes in the roots, root shoot junction, and shoot regions of rice seedlings under gravistimulation. Our results revealed that *OsPIN3a* and *OsPIN2* play a crucial role in role in rice simultaneously exposed to UV-C radiation, phytohormone treatment, and gravistimulation. Therefore, *OsPIN3a* and *OsPIN2* play a critical role in the plant defensive system against environmental threats (Fig. 7c–e). Similarly, we also demonstrated that high expression of *OsPIN1* might play an essential role in plant growth and development (Figs. 6a,b, and 3b).

## Conclusion

This study describes the effect of normal and artificial gravity on the amino acid profile, growth pattern, and expression of *OsPIN* genes in rice (*O. sativa* cv. Nagdong). Gravistimulation conditions were produced through clinostat rotation and by normal gravity (i.e., by changing the orientations of Petri dishes). As a result, the rice seedlings showed enhanced amino acids concentration, root and shoot growth, and expression of *OsPIN* genes versus controls. We also studied the gravistimulation effect combined with phytohormone treatments and UV-C radiation. These treatments differentially enhanced plant growth and *OsPIN* gene expression. We conclude that *OsPIN1* genes are highly induced by clinorotation and exogenously applied IAA and PAA plant hormones, that it might be responsible for enhancing the plant growth. Conversely, the *OsPIN2* and *OsPIN3a* genes, which act as gravity sensors and are induced in the root and root shoot junction of vertical and 90°-rotated seedlings under normal gravity, as well as in the UV-C stress condition, were mainly regulated by exogenously applied phytohormones JA, GA and 6-BAP. Based on our results, we conclude that vertical and 90°-rotated orientations under normal gravity and clinostat rotation can positively affect the biochemical composition of plant tissue. Therefore, these techniques could ultimately be productive in the agricultural sector, as well as for space exploration to enhance plant growth.
